# Usefulness of polymerase chain reaction for diagnosing Whipple’s disease in rheumatology

**DOI:** 10.1371/journal.pone.0200645

**Published:** 2018-07-18

**Authors:** Marion Herbette, Jean Baptiste Cren, Laurie Joffres, Charlotte Lucas, Emilie Ricard, Carine Salliot, Jérôme Guinard, Aleth Perdriger, Elisabeth Solau-Gervais, Béatrice Bouvard, Alain Saraux, on behalf of the Société de Rhumatologie de l’Ouest and the network VICTOR HUGO

**Affiliations:** 1 Rheumatology Unit, Hôpital de la Cavale Blanche, Brest, France; 2 Rheumatology Unit, CHU d’Angers,Angers, France; 3 Rheumatology Unit CHRU de Poitiers,Poitiers, France; 4 Rheumatology Unit, CHU, Hôpital Sud, Rennes, France; 5 Rheumatology and bacteriology Units, CHR, Orléans, France; 6 UMR1227, Lymphocytes B et Autoimmunité, Université de Brest, LabEx IGO, Brest, France; University College London, UNITED KINGDOM

## Abstract

**Objectives:**

To determine when *Tropheryma whipplei* polymerase chain reaction (PCR) is appropriate in patients evaluated for rheumatological symptoms.

**Methods:**

In a retrospective observational study done in rheumatology units of five hospitals, we assessed the clinical and radiological signs that prompted *T*. *whipplei* PCR testing between 2010 and 2014, the proportion of patients diagnosed with Whipple’s disease, the number of tests performed and the number of diagnoses according to the number of tests, the patterns of Whipple’s disease, and the treatments used. Diagnostic ascertainment was based on 1- Presence of at least one suggestive clinical finding; 2- at least one positive PCR test, and 3- a response to antibiotic therapy described by the physician as dramatic, including normalization of C Reactive Protein.

**Results:**

At least one PCR test was performed in each of 267 patients. Rheumatic signs were peripheral arthralgia (n = 239, 89%), peripheral arthritis (n = 173, 65%), and inflammatory back pain (n = 85, 32%). Whipple’s disease was diagnosed in 13 patients (4.9%). The more frequently positive tests were saliva and stool. In the centres with no diagnoses of Whipple’s disease, arthritis was less common and constitutional symptoms more common. The group with Whipple’s disease had a higher proportion of males, older age, and greater frequency of arthritis. The annual incidence ranged across centres from 0 to 3.6/100000 inhabitants.

**Conclusion:**

Males aged 40–75 years with unexplained intermittent seronegative peripheral polyarthritis, including those without constitutional symptoms, should have *T*. *whipplei* PCR tests on saliva, stool and, if possible, joint fluid.

## Introduction

Whipple’s disease is a rare chronic infection first described in 1907. The causative agent is the intracellular Gram-positive bacterium *Tropheryma whipplei*, which was identified by molecular sequencing in 1992[1]. This ubiquitous commensal organism very rarely causes disease. In the general population, polymerase chain reaction (PCR) screening of stool and saliva specimens showed a prevalence of healthy carriers of 1.5% to 7% and 0.2% to 1.5%, respectively[2].

In a study including 113 patients with systemic Whipple's disease in southern France, the main clinical symptoms were weight loss (n = 89, 79%), arthralgia (n = 88, 78%), and gastrointestinal disturbances (n = 80, 71%)[3]. Whipple's disease should be considered in patients with recurrent episodes of unexplained seronegative arthritis in the large limb joints, chronic diarrhoea, persistent fever, unexplained neurological signs, uveitis, blood-culture-negative endocarditis, and epithelioid granuloma[4,5]. Laboratory tests may show evidence of malabsorption, eosinophilia, erythrocyte sedimentation rate (ESR) and C-reactive protein (CRP) elevation, anaemia, thrombocytosis, and lymphopenia. None of these findings is specific. In some cases, the introduction of biotherapy after a mistaken diagnosis of rheumatoid arthritis or spondyloarthritis exacerbated the manifestations of Whipple’s disease[6,7].

Whipple's disease is too rare to warrant routine PCR screening in patients with polyarthritis or oligoarthritis[8]. Positive PCR testing of saliva, stool, and joint fluid has a good positive predictive value for Whipple’s disease[9,10,11]. However, duodenal biopsy remains the reference standard for diagnosing classic Whipple’s disease.

Several years usually elapse between symptom onset and the diagnosis. The mean time to diagnosis varies with the nature of the presenting symptoms, being longer in patients with joint manifestations (72 months) compared to those with gastrointestinal (48 months), constitutional (22 months), or neurological (30 months) manifestations[12]. The wide spectrum of clinical patterns raises diagnostic challenges. No consensus exists about the combination of clinical, laboratory, and radiological findings that warrant tests for Whipple's disease[1].

The primary aim of this multicentre retrospective study was to determine when patients evaluated for rheumatological symptoms should undergo *T*. *whipplei* PCR testing. Secondary aims were to describe the clinical patterns and treatments used, to determine the diagnostic yield of PCR testing, and to assess whether centres with higher numbers of tests also had a larger number of Whipple’s disease diagnoses.

## Methods

We conducted a retrospective observational study. The rheumatology units of the main hospital of each of five regions of western France (Brest, Angers, Poitiers, Orléans, and Rennes; see S1 Fig) identified all *T*. *whipplei* PCR tests and period acid-Schiff (PAS) stains performed to look for Whipple’s disease between 1 January 2010 and 31 December 2014. All patients were contacted by their physicians who signed a non opposition form. All data were fully anonymized. The protocol was approved by the appropriate ethics committee (Comité d’éthique du CHU de Brest; 2017CE19).

### PCR tests

All PCR tests for *T*. *whipplei* were performed at the bacteriology laboratory of the Marseille teaching hospital (Prof. Raoult). Real-time quantitative PCR tests targeting three repeated sequences of the *T*. *whipplei* genome was used. When an amplified product was detected, sequencing was performed and the results were routinely confirmed by a second PCR with a second set of primer pairs on the same sample [10]. We collected the nature of the tested samples (saliva, stool, blood, duodenal biopsy, urine, joint fluid, cerebro-spinal fluid, lung biopsy, cardiac valve, skin biopsy, ascites, disco-vertebral biopsy, or muscle biopsy).

### Clinical features of the tested patients

For each of the patients who had at least one *T*. *whipplei* PCR test, a physician (MH, LJ, JBC, ER, or CL) completed a standardized case-report form by abstracting the epidemiological, clinical, and radiological data from the medical charts.

### Patients diagnosed with Whipple’s disease

All charts of patients tested by *T*. *whipplei* PCR and diagnosed with Whipple’s disease at any of the participating hospitals were reviewed. All patients had at least one suggestive clinical finding; at least one positive PCR test, and a response to antibiotic therapy described by the physician as dramatic, including normalization of C Reactive Protein. We collected data on clinical, laboratory, and radiological findings and on treatments used.

The patients were divided into three groups depending on the type of Whipple’s disease: classic Whipple’s disease, defined as duodenal biopsy positive by PAS staining or *T*. *whipplei* immunohistochemistry, or as stool and saliva positive by PCR plus skin biopsy positive, or as blood positive by PCR; focal Whipple’s disease defined as joint fluid positive by PCR but duodenal biopsy negative by PAS staining and immunohistochemistry; or chronic *T*. *whipplei*-associated arthritis defined as chronic arthritis plus duodenal biopsy, stool, or saliva positive by PCR but duodenal biopsy negative by PAS staining and immunohistochemistry and joint fluid negative by PCR.

### Statistics

The data were keyboarded then analysed using the Statistical Package for the Social Sciences (SPSS 23.0, Chicago, IL). We first compared the populations tested by PCR across the five centres. We then compared the centres where Whipple’s disease was diagnosed to the other centres. Second, we described the samples tested by PCR in the patients diagnosed with Whipple’s disease. Third, we compared the characteristics and clinical features of patients with versus without a diagnosis of Whipple’s disease. Fourth, we determined the annual incidence of newly diagnosed Whipple’s disease in the study region.

Associations between study variables were assessed by univariate analysis using the chi-square test (or Fisher's exact test where appropriate) and the Mann-Whitney test. Variables yielding *p* values smaller than 0.05 were considered significant.

## Results

### Population tested by *T*. *whipplei* PCR

Over the five-year period, 267 patients had at least one *T*. *whipplei* PCR at the five rheumatology units. There were 139 women (52%) and 128 men with a mean age of 51.8 years and an age range of 15–84 years. The number of tested patients increased from each year to the next, from 20 in 2010 to 92 in 2014. Whipple’s disease was diagnosed in 13 patients (4.9%) (Table 1, S1 Fig).

**Table 1:** Tests that confirmed the diagnosis of Whipple’s disease (13 patients).

CASE	PAS on duodenal biopsy	PCR on stool	PCR on saliva	PCR on duodenal biopsy	PCR on joint fluid	PCR on blood	Pattern of Whipple disease
**BREST**							
**1**	negative	**positive**	**positive**	negative	not made	not made	CTWAA
**2**	negative	**positive**	**positive**	negative	not made	negative	CTWAA
**3**	negative	**positive**	**positive**	negative	**positive**	negative	FWD
**4**	**positive**	**positive**	**positive**	**positive**	not made	negative	CWD
**5**	negative	negative	negative	negative	**positive**	negative	FWD
**6**	negative	**positive**	negative	negative	not made	negative	CTWAA
**7**	negative	**positive**	**positive**	**positive**	not made	negative	CTWAA
**ANGERS**							
**8**	negative	**positive**	**positive**	**positive**	not made	negative	CWD
**9**	**positive**	**positive**	**positive**	**positive**	not made	**positive**	CWD
**10**	negative	**positive**	**positive**	not made	not made	negative	CTWAA
**11**	negative	**positive**	**positive**	not made	not made	negative	CTWAA
**12**	negative	**positive**	negative	negative	negative	negative	CTWAA
**13**	negative	**positive**	**positive**	**positive**	negative	negative	CWD

PAS, periodic acid-Schiff stain; PCR, polymerase chain reaction test for *Tropheryma whipplei*; CTWAA, chronic *Tropheryma whipplei*-associated arthritis; FWD, focal Whipple’s disease defined as joint fluid positive by PCR with duodenal biopsy negative by PAS and/or immunohistochemistry; CWD, classic Whipple’s disease defined as duodenal biopsy positive by PAS and/or immunohistochemistry or as stool and saliva positive by PCR plus skin biopsy or blood positive by PCR

### Symptoms that prompted PCR testing

The rheumatic symptoms that prompted PCR testing were peripheral arthralgia in 239 (89%) patients, peripheral arthritis in 173 (65%) patients, and inflammatory back pain in 85 (32%) patients. Radiological erosions were seen in 106 (39.7%) patients. Extra-articular manifestations were constitutional symptoms (n = 111, 41.8%), diarrhoea (n = 70, 26.5%), fever (n = 53, 20%), neurological signs (n = 11, 4.2%), uveitis (n = 7, 2%), lymphadenopathy (n = 4, 5.3%), pleural effusion (n = 2, 0.8%), and endocarditis (n = 1; 0.4%).

Differences were found across centres for the following reasons for PCR testing: arthritis, radiological erosions, constitutional symptoms, fever, diarrhoea, lymphadenopathy, and neurological signs (S1 Table). Differences in gender of tested patients were also found. In the two centres with diagnoses of Whipple’s disease, the PCR-tested population had more patients with arthritis but fewer patients with constitutional symptoms or fever compared to the three other centres. These two centres also had a larger number of PCR tests on stool samples than did the other centres (Table 2).

**Table 2:** Comparison of patients tested at the two centres with diagnoses of Whipple’s disease (Brest and Angers) to patients tested at the other three centres (Orléans, Poitiers and Rennes).

*Clinical signs*	Brest and Angers:13 diagnoses161 tests	Orléans, Poitiers, and Rennes: no diagnoses106 tests	*p* value univariate*	*p* value multivariate**
**Age**	53.42 (14.75)	49.30 (15.80)	**0.05**	**0.029**
**Male gender**	82/161 (51.0)	46/106 (43.4)	0.23	0.11
**Arthralgia**	144/161 (89.4)	95/106 (89.6)	0.96	
**Arthritis**	120/161 (74.5)	53/106 (50.0)	**<0.001**	**<0.001**
**Radiological erosion**	65/161 (40.4)	43/106 (40.6)	0.97	
**Inflammatory lowback pain**	49/161 (30.4)	36/106 (34.0)	0.54	
**Constitutional symptoms**	57/161 (35.4)	54/105 (51.4)	**0.01**	**0.016**
**Fever**	22/160 (13.7)	31/106 (29.2)	**0.002**	**0.001**
**Diarrhoea**	38/161 (23.6)	32/103 (31.1)	0.18	0.103
**Lymphadenopathy**	5/161 (3.1)	9/101 (8.9)	**0.04**	0.066
**Uveitis**	5/161 (3.1)	2/91 (2.2)	0.67	
**Neurological signs**	4/161 (2.5)	7/101 (6.9)	0.08	0.066
**Pleural effusion**	0/160 (0)	2/100 (2)	0.07	0.066
**Endocarditis**	0/161 (0)	1/106 (0.9)	0.22	0.195
***Tests performed in centers***				
**Stool PCR**	134/161 (83.2)	41/106 (38.7)	**<0.0001**	
**Saliva PCR**	145/161 (90.1)	65/106 (61.3)	**<0.0001**	
**Joint fluid PCR**	41/161 (25.5)	9/106 (8.5)	**0.0005**	
**Duodenal biopsy**	46/161 (28.6)	30/106 (28.3)	0.96	
**Urine PCR**	57/161 (35.4)	12/106 (11.3)	**<0.0001**	
**Blood PCR**	93/161 (57.8)	71/106 (67.0)	0.13	
**CSF PCR**	5 /161 (3.1)	2/106 (1.9)	0.54	

The data are number of patients with the symptom or positive PCR test over total number (%) of patients with information on the symptom or PCR test.

PCR, polymerase chain reaction for *Tropheryma whipplei*; CSF, cerebrospinal fluid.

*Khi2 (or Fisher's exact test where appropriate) for dichotomous data.

** Logistic regression.

### Samples tested by *T*. *whipplei* PCR

The three main sample types used for PCR testing were saliva (n = 210, 78.9%), stool (n = 175, 65.5%), and blood (61.4%; n = 164/267),

The distribution of positive PCR tests across centres differed between stool samples (Table 3).

**Table 3:** Distribution of positive PCR tests for *Tropheryma whipplei* by centre and sample type.

	Centres with diagnoses of Whipple’s disease		Centres without diagnoses of Whipple’s disease			
	Angers	Brest	Orléans	Poitiers	Rennes	*p* value*
**Stool**	11/60 (18.3)	9/74 (12.2)	0/25 (0)	0/16 (0)	0/0 (0)	**0.04**
**Saliva**	6/60 (10)	5/85 (5.9)	0/24 (0)	1/20 (5.0)	1/21(4.8)	0.52
**Joint fluid**	0/13 (0)	3/28 (10.7)	0/2 (0)	0/2 (0)	0/2 (0)	0.64
**Duodenal biopsy**	3/20 (15)	1/26 (3.8)	0/17 (0)	0/0 (0)	0/13(0)	0.13
**Blood**	1/57(1.7)	0/36 (0)	0/19 (0)	0/46 (0)	0/6 (0)	0.75

The data are number of patients with the test over total number of patients (%) with information on the test.

CSF, cerebrospinal fluid.

*Khi 2 (or Fisher's exact test where appropriate).

### Population diagnosed with Whipple’s disease and healthy carriers

**Fig 1:**
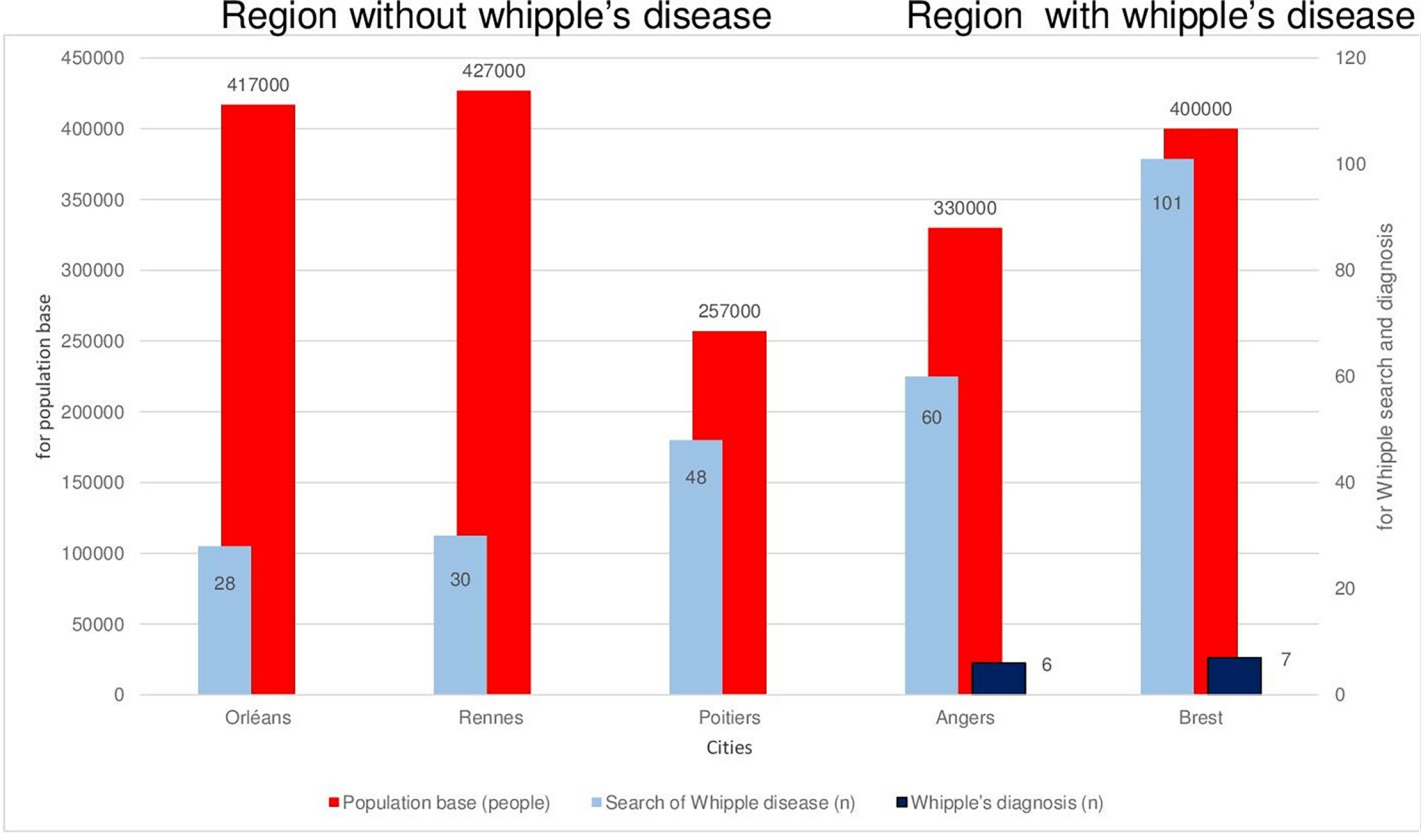
Number of patients with at least one PCR test (sky blue) and with a diagnosis of Whipple’s disease (deep blue) according to population in each of the five regions of western France (red).

Whipple’s disease was diagnosed in 13 (4.9%) of the PCR-tested patients, one in 2010, three in 2011, five in 2012, one in 2013, and three in 2014. All diagnoses were made at two of the five centres, seven in Brest and six in Angers, where the numbers of tested patients were 101 (7% positive), and 60 (10% positive), respectively. No cases were diagnosed in Orleans, Poitiers, or Rennes, where the numbers of tested patients were 28, 48, and 30, respectively (Fig 1, S1 Fig).

Of the 13 patients, four (30.8%) had classic Whipple’s disease, two (15.4%) focal Whipple’s disease, and seven (53.8%) chronic *T*. *whipplei*-associated arthritis.

Among patients diagnosed with Whipple’s disease, the most common combination of positive PCR tests was saliva, stool, and duodenal biopsy or joint fluid (n = 7, 53.8%). PCR on stool samples was performed more often, and was more often positive, in the centres with than without diagnoses of Whipple’s disease (Table 4).

**Table 4:** Comparison tested patients with and without a diagnosis of Whipple’s disease.

	Patients with Whipple disease, n = 13	Patient without Whipple disease, n = 254	*p* value*	*p* value**
**Age, years (SD)**	60.5 (11.1)	51.3 (15.35)	**0.03**	0.094
**Male gender**	11/13 (84.6)	117/254 (46.1)	**0.007**	**0.019**
**Arthralgia**	13/13 (100)	226/254 (89)	0.21	0.998
**Arthritis**	13/13 (100)	160/254 (63)	**0.01**	0.996
**Inflammatory low back pain**	3/13 (23.1)	82/254 (33.3)	0.49	
**Constitutional symptoms**	6/13 (46.1)	105/253 (41.5)	0.74	
**Fever**	3/13 (23.1)	50/253 (19.8)	0.72	
**Diarrhoea**	3/13 (23.1)	67/251 (26.7)	0.77	
**Lymphadenopathy**	0/13 (0)	14/249 (5.6)	0.38	
**Uveitis**	0/13 (0)	7/239 (2.9)	0.53	
**Neurological signs**	0/13 (0)	11/249 (4.4)	0.44	
**Endocarditis**	0/13 (0)	1/254 (0.4)	0.75	
**Pleural effusion**	0/13 (0)	2/247 (0.8)	0.74	
**Radiological erosions**	6/13 (4.6)	102/254 (40.2)	0.67	
**Stool PCR positive**	12/13 (92.3)	8/162 (4.9)	**<0.001**	
**Saliva PCR positive**	10/13 (77.0)	3/197 (1.5)	**<0.001**	
**Joint fluid PCR positive**	2/4 (50.0)	1/46 (2.2)	**<0.001**	
**Duodenal biopsy PCR positive**	4/9 (44.4)	0/67 (0)	**<0.001**	
**Urine PCR positive**	1/7 (14.3)	0/62 (0)	0.11	
**Blood PCR positive**	1/12 (8.3)	0/152 (0)	0.07	
**CSF PCR positive**	0/3 (0)	0/4 (0)	-	

The data are number of patients with the symptom or PCR test positive over total number of patients (%) with information on the symptom or PCR test.

PCR, polymerase chain reaction for *Trophyrema whipplei*; CSF, cerebrospinal fluid.

*Khi2 (or Fisher's exact test where appropriate).

** Logistic regression.

Of the 267 tested patients, 11 (4.1%) were healthy carriers, with an isolated positive PCR test on saliva (3/267) or stool (8/267).

### Proportion of diagnoses according to patient features

The group with Whipple’s disease had a higher proportion of males, older age, and greater frequency of arthritis compared to the group without Whipple’s disease (Table 4). In the centres with no diagnoses of Whipple’s disease, arthritis was less common, whereas constitutional symptoms, fever, and lymphadenopathy were more common (Table 2). The features with the best sensitivity were male gender, age 40–75, and arthritis but only male gender was associated with the diagnosis of Whipple’s disease by multivariate analysis (Table 4) and no feature had both good sensitivity and specificity (Table 5). The combination of the 3 tests (male gender, age 40–75, and arthritis) obtained a positive predictive value of 15.2% and a negative predictive value of 98.5%.

**Table 5:** Diagnostic value of demographical, clinical and radiological features.

	TP	FP	FN	TN	Se	Sp	PPV	NPV
**Male gender**	11	117	2	137	84.6	53.9	8.6	98.6
**Arthralgia**	13	226	0	28	100	11.0	5.4	100
**Arthritis**	13	160	0	94	100	37.0	7.5	100
**Inflammatory low back pain**	3	80	10	172	23.1	67.7	3.5	94.5
**Constitutional symptoms**	6	105	7	148	46.1	58.5	5.4	95.5
**Fever**	3	50	10	203	23.1	80.2	5.7	95.3
**Diarrhoea**	3	67	10	184	23.1	73.3	4.3	94.8
**Lymphadenopathy**	0	14	13	137	0	94.4	0	94.8
**Radiological erosions**	6	102	7	152	25.0	59.8	5.6	95.6
**Male gender and age 40–75 and arthritis**	10	56	3	198	76.9	77.9	15.2	98.5

The data are number of patients in the first four columns and percentages in the last four columns.

TP, true positive; FP, false positive; FN, false negative; TN, true negative (TN); Se, sensitivity; Sp, specificity; PPV, positive predictive value; NPV, negative predictive value.

Finally, the best way to diagnose Whipple’s disease is the two steps combination male gender, age 40–75, arthritis, and at least one stool or saliva positive PCR (S2 Fig).

### Proportion of diagnoses across the five study regions

The total population in the five study regions was 1 831 000 (https://www.insee.fr/fr/statistiques). With 13 cases over five years, the annual incidence was 1.4 per million. However, the annual incidence varied across regions from 0 to 3.6/100 000; it was highest in Angers (3.6/100 000), followed by Brest (3.5/100 000), which were the two centres with the highest number of PCR tests and the only two centres with diagnoses of Whipple’s disease. The higher number of tests in the two centres with diagnoses was associated to both a higher number of tested patients and a higher number of samples tested per patient.

### Characteristics of patients diagnosed with Whipple’s disease

Of the 13 patients diagnosed with Whipple’s disease, 11 (84.6%) were males. Median age was 61.2 years (range, 42–75 years). Only one patient was farmer and none of the remainders had contacts with animals.

Before the diagnosis of Whipple disease, 12 of the 13 patients were misdiagnosed with one of the following inflammatory joint diseases: unexplained polyarthritis (n = 4), rheumatoid arthritis (n = 3), spondyloarthritis with sacroiliitis (n = 3), sarcoidosis (n = 1), and psoriatic arthritis (n = 1). Mean time from symptom onset to the diagnosis of Whipple’s disease was 7.7 years (range, 1–17 years). Immunosuppressants had been used in eight (61.5%) patients, including corticosteroids (n = 5, 38.5%), disease-modifying anti-rheumatic drugs (n = 5, 38.5%), and TNFα antagonists (n = 2, 15.4%). The two patients given TNFα antagonist therapy received one and two courses, respectively, and were not reported to have experienced symptom exacerbation during the treatment.

All 13 patients diagnosed with Whipple’s disease had arthritis and eight (61.5%) had polyarthritis. The joint manifestations were usually acute and intermittent (n = 9, 69.2%) and non-erosive (n = 10, 77.0%). In some patients, the picture mimicked rheumatoid arthritis with involvement of the wrist (S3 Fig). The affected joints were the knees (n = 8, 61.5%), ankles (n = 5, 38.5%), wrists (n = 5, 38.5%), small hand joints (n = 4, 30.8%), small foot joints (n = 2, 15.4%), shoulders (n = 2, 15.4%), and elbows (n = 1, 7.7%). One patient had multiple discitis (S4 Fig). Of the five patients who underwent sacro-iliac radiography or magnetic resonance imaging, three had sacro-iliitis.

Extra-articular manifestations were constitutional symptoms (n = 8, 61.5%) and fever (n = 3, 23.1%). Only two (15.4%) patients had diarrhoea and one (7.7%) pericarditis. No patient had neurological signs, abdominal pain, lymphadenopathy, uveitis, pleural effusion, or endocarditis.

The main findings from standard blood tests were CRP elevation (n = 11, 84.6%), ESR elevation (n = 9, 69.2%), serum fibrinogen elevation (n = 7, 70.0%), and anaemia (n = 5, 38.5%). A few patients had leucocytosis (n = 4, 30.8%) or thrombocytosis (n = 2, 15.4%). A single patient had positive tests for rheumatoid factors. In none of the patients were tests positive for anti-citrullinated peptide antibodies, anti-neutrophil cytoplasmic antibodies, or HLA B27.

In two patients with arthritis, the diagnosis of Whipple’s disease was considered when antibiotic therapy for cholecystitis or erysipelas was followed by dramatic improvements in the joint manifestations and laboratory signs of systemic inflammation.

*T*. *whipplei* PCR was positive in at least two sample types in 10 (77.0%) patients and in a single sample type in three (23.1%) patients (Table 1). The duodenal biopsy was positive by PAS staining in two (15.4%) patients, both of whom also had at least one positive PCR test. The sample types most often positive by PCR were stool (12/13) and saliva (10/13), followed by duodenal biopsy (4/9); less often, PCR was positive in joint fluid (2/4), urine (1/7), and/or blood (1/13). None of cerebrospinal fluid samples were positive.

The first-line treatment of Whipple’s disease in all patients was hydroxychloroquine (400 mg/day [4/13, 30.8%] or 600 mg/day [8/13, 61.5%]) combined with doxycycline (200 mg/day). The antibiotic was changed to intravenous ceftriaxone after one month for one week in one patient and to trimethoprim-sulfamethoxazole after one month for another patient (and then hydroxychloroquine combined with doxycycline was ordered again). Mean hydroxychloroquine treatment duration was 19.3 months (range, 12–24 months). Mean antibiotic treatment duration was 19.6 months in nine (69.2%) patients; the remaining four patients are still taking an antibiotic. The treatment was consistently effective in resolving the clinical and laboratory abnormalities. Follow-up *T*. *whipplei* tests were done despite the absence of evidence of recurrence in eight (58.3%) patients and were consistently negative. In two (15.4%) patients, treatment discontinuation was followed by a recurrence of the inflammatory joint symptoms after 18 and 24 months, respectively, which was treated with hydroxychloroquine and doxycycline. In one patient with recurrent symptoms the symptoms persisted despite negative PCR tests and were ascribed to unexplained chronic joint disease, possibly post infectious in nature.

## Discussion

In western France, the incidence of Whipple’s disease newly diagnosed in rheumatology departments varied across regions and showed a strong association with the number of *T*. *whipplei* PCR tests performed.

Our findings may have important clinical implications. Chronic *T*. *whipplei*-associated arthritis was more common than classic and focal Whipple’s disease. This finding suggests that classic Whipple’s disease may contribute only a small proportion of cases with rheumatic manifestations. These last may occur chiefly when non-invasive *T*. *whipplei* proliferation in the gut induces reactive arthritis, with joint-fluid PCR results that are either positive (focal Whipple’s disease) or negative (*T*. *whipplei*-associated arthritis). Therefore, for patients with rheumatic manifestations, the diagnostic strategy suggested by gastroenterologists may not be optimal. In gastroenterology patients, the first-line investigation is duodenoscopy with multiple duodenal biopsies for PAS-staining, PCR, and/or immunohistochemistry[13]. Our data suggest that, in rheumatology patients, PCR tests should be performed first, on saliva and stool samples and, when arthritis is present, on joint fluid samples, as joint-fluid PCR seems highly specific[6,14]. The results of these first-line tests should be interpreted in the light of the clinical setting (number of signs suggesting Whipple’s disease, number of different samples positive by PCR, and whether antibiotic therapy given for another reason was followed by a dramatic improvement). Second-line tests may then be selected on a case-by-case basis; they may include PCR testing of blood samples and duodenoscopy with multiple biopsies. Tests for neurological involvement are indicated in patients with classic Whipple’s disease[15,16] but may be less useful in those with the other two disease patterns. A dramatic response to appropriate treatment was seen consistently in our study, providing further support for the diagnosis.

The manifestations prompting PCR testing in our study is limited. We were unable to identify a combination of manifestations that should lead with a perfect balance of sensitivity and specificity to PCR testing. Age 40–75, male gender and arthritis were finally the signs most associated to the disease. Constitutional symptoms and fever were sensitive manifestation but all three are non-specific. Given the absence of sensitive and specific clinical manifestations, increasing the number of PCR tests and the numbers of samples tested per patient done to investigate unexplained arthritis may be the best means of diagnosing Whipple’s disease. The number of patients tested increased over time in our study, reflecting progress in the knowledge of *T*. *whipplei*. The proportion of positive tests remained stable at about 4% in another study [17] but this proportion is clearly different from a hospital to another in our study. We suggest to limit PCR to males aged 40–75 years with unexplained intermittent seronegative peripheral polyarthritis, including those without constitutional symptoms, as we obtained a positive predictive value of 15.2 for a negative predictive value of 98.5 using the combination of these three criteria in our study. Three woman had Whipple’s disease justifying to do PCR in some cases but probably less systematically than in male with unexplained arthritis.

The considerable variability in the incidence of diagnosed Whipple’s disease across regions in our study may be due to differences in the number of tests performed rather than to differences in the incidence of the disease. All five regions are located in western France, where variations in the environmental reservoir of *T*. *whipplei* are probably limited[18]. Performing a large number of PCR tests in male patients with suggestive rheumatic manifestations (at least seronegative arthritis) can be expected to improve the proportion of diagnosed cases[19].

Limitations of our study include the small number of patients diagnosed with Whipple’s disease. However, Whipple’s disease is rare. Furthermore, the criteria used to classify some of the patients into the non-classical of Whipple’s disease groups may deserve discussion.

In conclusion, Whipple’s disease with rheumatic manifestations is rare. Greater use of PCR testing of stool and saliva samples was associated with diagnosing Whipple’s disease. The typical patient is a rheumatoid factor-negative male with chronic intermittent polyarthritis of the large joints [6,20]. Inflammatory low back pain with a negative test for HLAB27 should also suggest the diagnosis. Gastrointestinal manifestations were present in only one-quarter of our rheumatology patients and consolidate the existence of joint localized Whipple’s disease [21] and Whipple’s associated arthritis.

## Supporting information

S1 Fig Location and population in the two regions where cases of Whipple’s disease were diagnosed (Brest and Angers, in red) and in the other three regions (Orléans, Poitiers, and Rennes, in blue). (TIF)

S2 Fig Diagnostic value of the combination clinical sign/PCR. (TIF)

S3 Fig Example of Whipple’s disease mimicking rheumatoid arthritis. (TIF)

S4 Fig Example of Whipple’s disease mimicking ankylosing spondylitis. (TIF)

S1 Table Comparison of the five centres regarding the clinical presentations in patients who underwent PCR testing. (DOCX)
