# Supplementary figures and images for: Usefulness of polymerase chain reaction for diagnosing Whipple’s disease in rheumatology
Source: PLoS One. 2018 Jul 18;13(7):e0200645. doi: 10.1371/journal.pone.0200645 (PMC6051605; doi:10.1371/journal.pone.0200645)

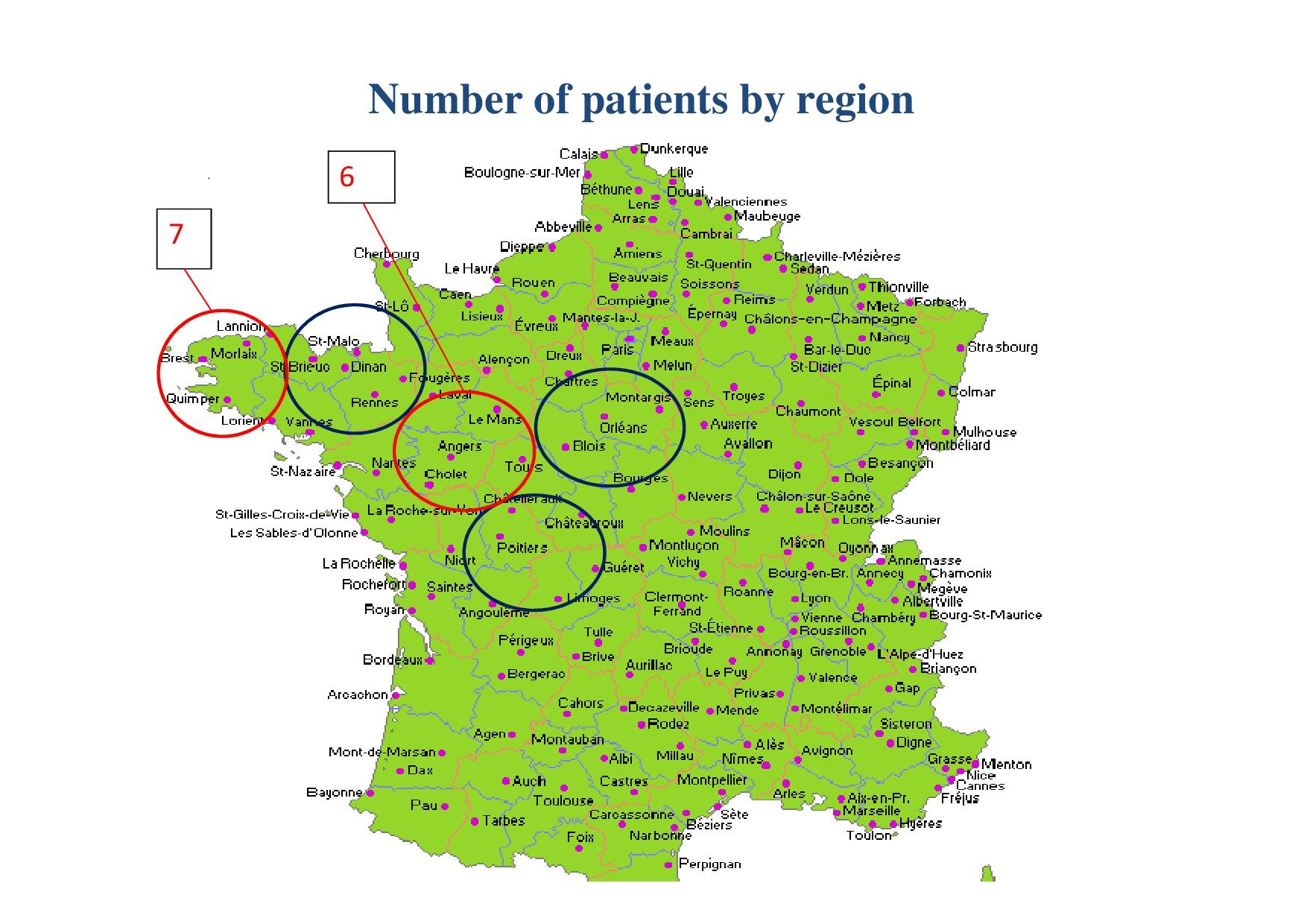

Supplement: S1 Fig — (TIF) [file pone.0200645.s001.tif]

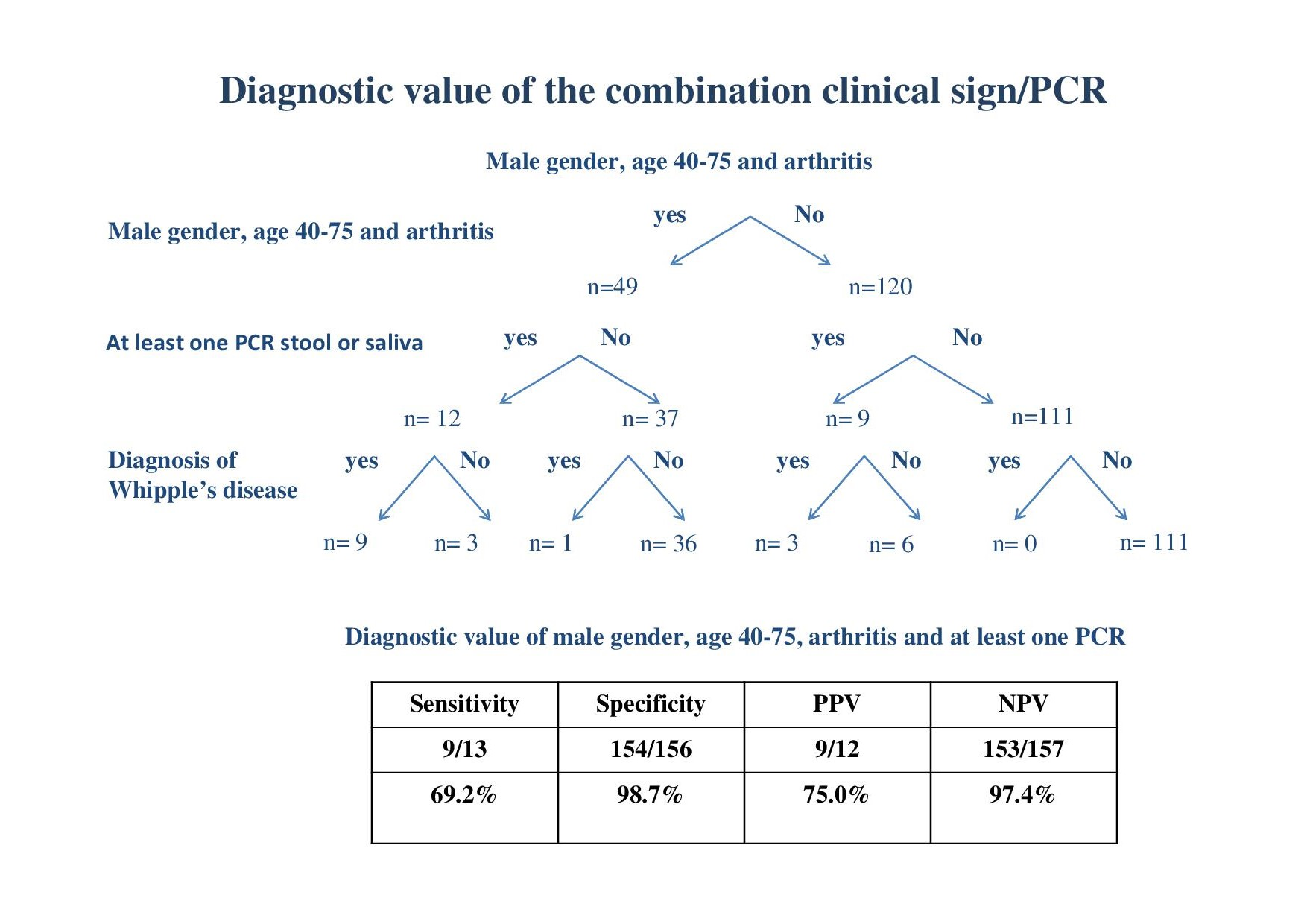

Supplement: S2 Fig — (TIF) [file pone.0200645.s002.tif]

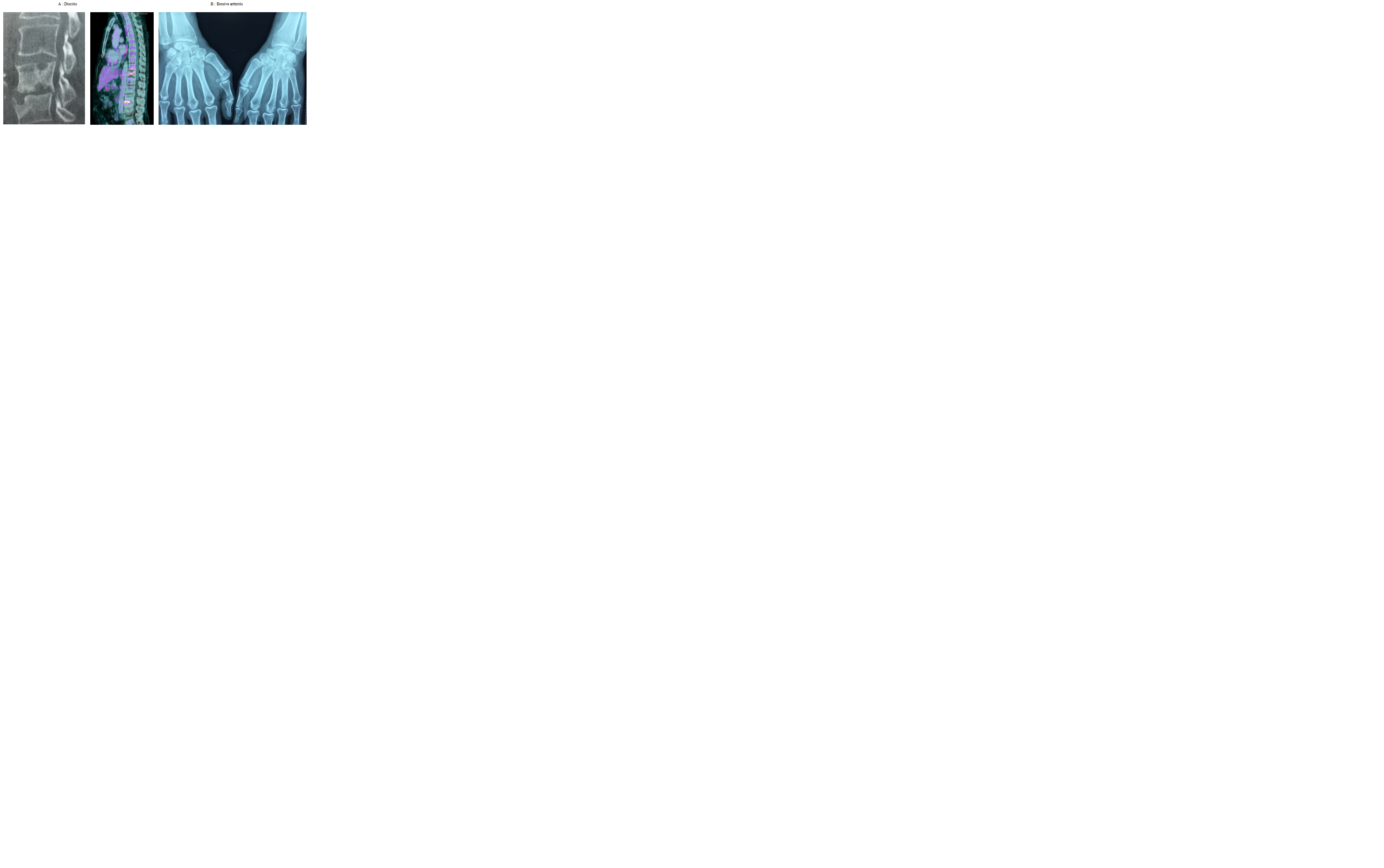

Supplement: S3 Fig — (TIF) [file pone.0200645.s003.tif]

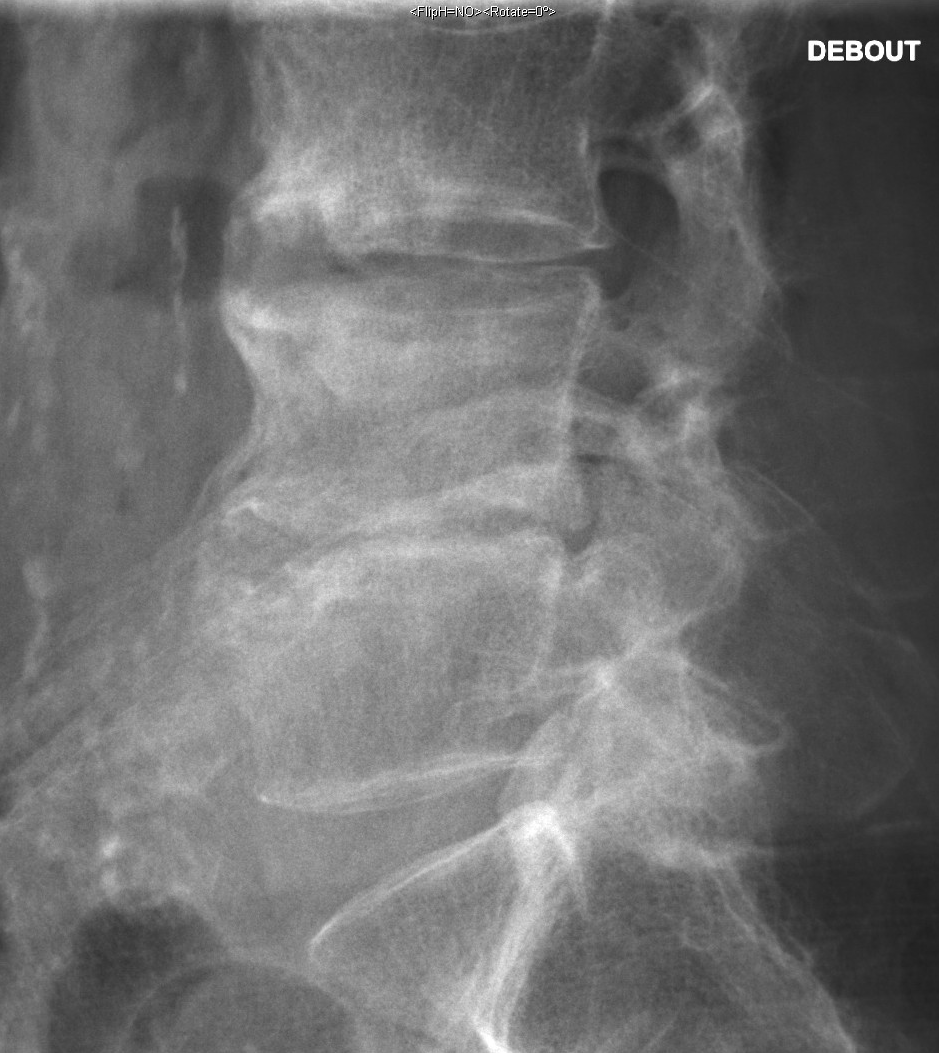

Supplement: S4 Fig — (TIF) [file pone.0200645.s004.tif]
