# Supplementary material for: Usefulness of polymerase chain reaction for diagnosing Whipple’s disease in rheumatology
Source: PLoS One. 2018 Jul 18;13(7):e0200645. doi: 10.1371/journal.pone.0200645 (PMC6051605; doi:10.1371/journal.pone.0200645)
Supplement: S1 Table — (DOCX) [file pone.0200645.s005.docx]

**S2 Table: Comparison of the five centres regarding the clinical presentations in patients who underwent PCR testing**

| Centre | Angers | Brest | Orléans | Poitiers | Rennes | *p* value* |
| --- | --- | --- | --- | --- | --- | --- |
| Male gender | 36/60 (60.0) | 46/101 (45.5) | 9/28 (32.1) | 16/48 (33.3) | 21/30 (70.0) | **0.002** |
| Arthralgia | 55/60 (91.7) | 89/101 (88.1) | 25/28 (89.3) | 42/48 (87.5) | 28/30 (93.3) | 0.88 |
| Arthritis | 55/60 (91.7) | 65/101 (64.4) | 10/28 (35.7) | 25/48 (52.1) | 18/30 (60.0) | **<0.001** |
| Radiological erosions | 41/63 (68.2) | 22/101 (21.8) | 0/28 (0) | 25/48 (52.1) | 18/30 (60.0) | **<0.001** |
| Inflammatory low back pain | 18/60 (30.0) | 31/101 (30.7) | 12/28 (42.9) | 12/48 (25.0) | 12/30 (40) | 0.45 |
| Constitutional symptoms | 11/60 (18.3) | 46/101 (45.5) | 18/28 (64.3) | 23/48 (47.9) | 13/29 (44.8) | **<0.001** |
| Fever | 4/60 (6.7) | 18/100 (18) | 12/28 (42.9) | 13/48 (27.1) | 6/30 (20.0) | **0.001** |
| Diarrhoea | 15/60 (25.0) | 23/101 (22.8) | 19/28 (67.9) | 11/48 (22.9) | 2/27 (7.4) | **<0.001** |
| Lymphadenopathy | 3/60 (5.0) | 2/101 (2.0) | 3/28 (10.7) | 1/48 (2.1) | 5/25 (20.0) | **0.004** |
| Uveitis | 1/60 (1.7) | 4/101 (4.0) | 0/28 (0) | 1/48 (2.1) | 1/15 (6.7) | 0.64 |
| Endocarditis | 0/60 (0) | 0/101 (0) | 1/28 (3.6) | 0/48 (0) | 0/48 (0) | 0.07 |
| Neurological signs | 0/60 (0) | 4/101 (4.0) | 4/28 (1.4) | 3/48 (6.2) | 0/25 (0) | **0.02** |
| Pleural effusion | 0/60 (0) | 0/100 (0) | 1/28 (3.6) | 1/48 (2.1) | 0/24 (0) | 0.25 |

The data are number of patients with the symptom over total number of patients (%) with information on the symptom.

*Khi2 (or Fisher's exact test where appropriate)
